# Orbital Metastasis from Hidradenocarcinoma

**DOI:** 10.22336/rjo.2025.46

**Published:** 2025

**Authors:** Mary Stephen, Kumaradarshini Maruthachalam, Nirupama Kasturi, Jayasri Periyandavan

**Affiliations:** Department of Ophthalmology, Jawaharlal Institute of Postgraduate Medical Education and Research, Puducherry, India

**Keywords:** hidradenocarcinoma, adnexal tumour, metastasis, proptosis, optic nerve involvement

## Abstract

Hidradenocarcinoma is a rare form of malignant adnexal tumor. This malignant tumor has been reported to metastasize to regional lymph nodes, and distant metastasis into structures like the orbit and extraocular muscle is uncommon. Orbital metastasis comprises about 10% of all orbital neoplasms. Common systemic malignancies contributing to orbital metastasis are breast cancer, prostate cancer, and skin malignancy, melanoma. We report the case of a middle-aged man with left axillary hidradenocarcinoma with an orbital metastatic lesion infiltrating the inferior rectus muscle with optic nerve involvement. This case report describes a rare presentation of orbital metastasis from an adnexal tumour.

## Introduction

Orbital metastases are rare, accounting for only two to four percent of all orbital space-occupying lesions. Solid tumors typically reach the orbit through the hematogenous route. The presence of orbital metastasis often signifies an advanced stage of the primary disease. While virtually any internal organ or skin cancer has the potential to metastasize to the orbit, the majority of orbital metastases are derived from lung, breast, and prostate cancers. Hidradenocarcinomas are rare, aggressive skin adnexal tumors originating from sweat glands and are associated with a significant risk of local recurrence and metastasis [1]. These tumors have a recurrence rate of up to 50%, even after aggressive surgical intervention. Although metastasis to lymph nodes, bones, vertebrae, and lungs has been well-documented, orbital metastasis has not been previously reported. In this report, we presented a case of orbital metastasis originating from cutaneous hidradenocarcinoma.

## Case report

**Fig. 1:**
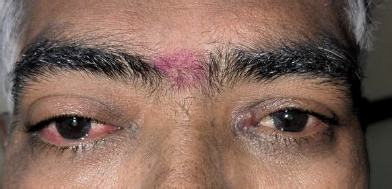
Clinical photograph of patient showing presence of inferior conjunctival chemosis with lid edema and prominence of right eye compared with left eye

**Fig. 2:**
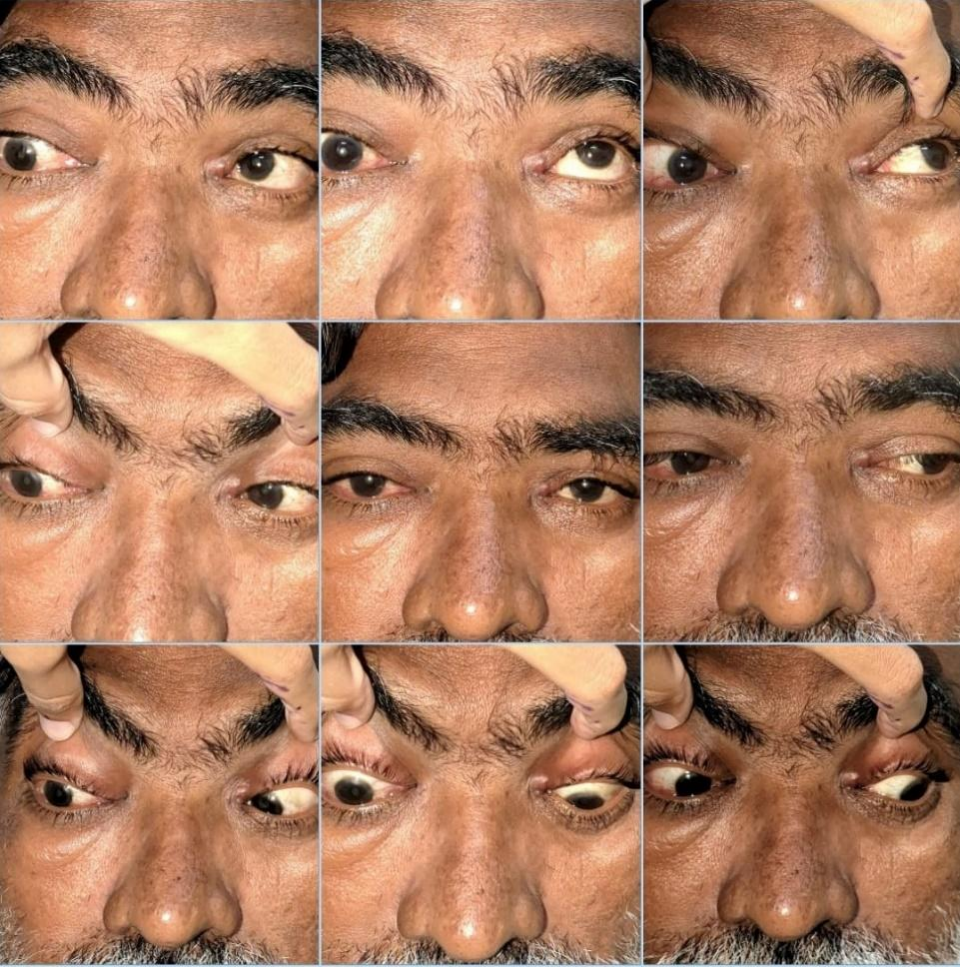
Nine-gaze photograph of the patient demonstrating restriction of right eye extraocular movements, with maximum restriction involving up gaze, with normal movements in the left eye

A 57-year-old male complained of redness, watering in the right eye, and diplopia on the upgaze for the past two weeks. He had a history of recurrent malignant hidradenocarcinoma of the left axilla and was scheduled for wide local excision. The primary tumor had been excised in 2018, followed by recurrence in 2022 with axillary vein infiltration, for which he underwent wide local excision and resection of the axillary vein, with a great saphenous vein graft. Visual acuity, by Snellen’s chart, was 6/6 in both eyes. Mild restriction of extraocular movements was noted, with more profound limitation in the right eye, and normal movements in the left eye. Minimal proptosis with chemosis and lid edema was found in the right eye (**Fig. 1, 2**).

**Fig. 3:**
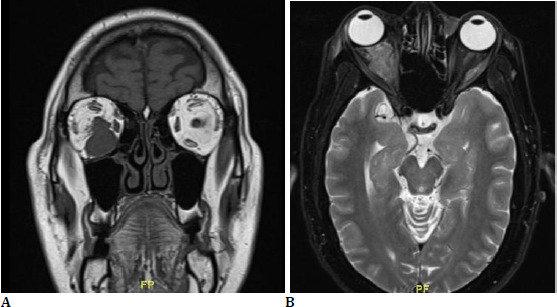
**A**. T1 MRI coronal section showing a hypointense lesion in the right eye replacing the inferior rectus muscle; **B**. Axial section showing irregular lesion with 180-degree sectoral contact with optic nerve

Intraocular pressure in the right eye was 36 mmHg on upgaze, 20 mmHg in primary gaze, and 14 mmHg on downgaze, with open angles on gonioscopy, and the left eye intraocular pressure was 12 mmHg. Hertel’s measurement using a 110 mm base was 22 mm (right eye) and 19 mm (left eye). Fundus examination revealed a hyperemic disc in the right eye, while the left eye was normal. Contrast-enhanced computed tomography imaging showed a lesion in the inferomedial aspect of the right orbit, displacing the globe superolaterally. The lesion was longitudinally oriented along the inferior rectus muscle. Magnetic resonance imaging revealed an irregular, hypointense lesion on T1-weighted images, located in the right inferior rectus, with 180-degree sectoral contact with the right optic nerve. The lesion exhibited heterogeneous post-contrast enhancement, suggesting the possibility of orbital metastasis (**Fig. 3**).

Additionally, multiple enhancing lesions were observed in the dura and the right temporal lobe, indicating metastatic involvement. The patient underwent radiotherapy in multiple sittings; however, they were lost to follow-up for subsequent visits.

## Discussion

Orbital metastasis is the most common malignant tumour in adults; however, rare metastatic cancers account for approximately 2% of all orbital tumors. The diagnosis of primary orbital malignancy is often straightforward and well-characterized; however, metastatic lesions in the orbit usually present with varied symptoms, ranging from subtle signs to severe lesions, posing diagnostic challenges. A specific range of cancers metastasize to the orbit, including hidradenocarcinoma. This adnexal malignancy is uncommon, making this case report of orbital metastasis from hidradenocarcinoma a noteworthy contribution to the understanding of this unusual clinical phenomenon. The apocrine sweat glands are the source of the fulminant cancer, hidradenocarcinoma. Although uncommon cases have been documented in anatomical sites, such as the breast and face, it most frequently appears in the skin, especially in the axillary, perineal, or anogenital regions [1]. With a high risk of distant metastases, especially to the liver, lungs, and bones, and a generally poor prognosis, hidradenocarcinoma is a fulminant metastatic tumour [2]. As demonstrated here, the orbit’s involvement is infrequent. Proptosis, discomfort, diplopia, and impaired visual acuity are common symptoms of orbital metastasis. These symptoms often overlap with those of other orbital illnesses, making diagnosis more difficult. This highlights the importance of considering metastatic lesions in the differential diagnosis of orbital masses [3]. Orbital metastases from hidradenocarcinoma are uncommon. Although the internal carotid artery supplies the majority of the orbit’s blood supply and provides a rich vascular environment, metastasis from cancers originating in the skin or soft tissues is not commonly reported [4]. Orbital metastases from hidradenocarcinoma have been reported in the literature, typically in advanced stages of the illness; however, spreading into orbital tissues, to our knowledge, is extremely rare [5]. These metastases can spread hematogenously or directly from nearby sites, such as the nasopharynx or paranasal sinuses [6]. The clinical management of orbital metastasis from hidradenocarcinoma generally follows the same principles as the treatment of other orbital metastases. Treatment is aimed at controlling local disease, alleviating symptoms, and preventing further metastasis. In this context, systemic therapy, including chemotherapy and targeted therapies, is often used to manage the primary tumor and prevent further metastatic spread [7]. Surgical intervention, such as debulking of the orbital mass, may be considered for palliative purposes if the tumor causes significant visual or cosmetic impairment. In some cases, radiotherapy may also be employed to reduce tumor burden and provide symptom relief [8].

## Conclusion

This case report emphasizes the importance of careful examination and highlights the detection of subtle signs in orbital tissues, particularly in patients with potential malignant lesions. While orbital metastasis from hidradenocarcinoma is exceedingly rare, its potential for occurrence underscores the need for comprehensive management strategies that incorporate multidisciplinary care. Prognosis in these cases is generally poor, with median survival ranging from months to a few years, depending on the extent of metastasis and response to treatment.

Further studies are warranted better to understand the mechanisms of metastasis to the orbit and to optimize therapeutic approaches for these patients.
